# Human oocyte microtubule organizing center: a newly identified driver for meiotic spindle assembly in female oocytes

**DOI:** 10.1093/lifemedi/lnad016

**Published:** 2023-04-26

**Authors:** Jie Dong, Tianyu Wu, Qing Sang, Lei Wang

**Affiliations:** Department of Reproductive Medical Center, Jinling Hospital, Nanjing University, School of Medicine, Nanjing 210002, China; Institute of Pediatrics, Children's Hospital of Fudan University, State Key Laboratory of Genetic Engineering, Institutes of Biomedical Sciences, Shanghai Key Laboratory of Medical Epigenetics, Fudan University, Shanghai 200032, China; Institute of Pediatrics, Children's Hospital of Fudan University, State Key Laboratory of Genetic Engineering, Institutes of Biomedical Sciences, Shanghai Key Laboratory of Medical Epigenetics, Fudan University, Shanghai 200032, China; Institute of Pediatrics, Children's Hospital of Fudan University, State Key Laboratory of Genetic Engineering, Institutes of Biomedical Sciences, Shanghai Key Laboratory of Medical Epigenetics, Fudan University, Shanghai 200032, China

The long-standing opinions view human oocytes as lacking a prominent microtubule organizing center (MTOC). However, a recent study published in *Science* by Wu et al. has identified a unique MTOC-like structure named human oocyte microtubule organizing center (huoMTOC) which is responsible for meiotic spindle assembly in human oocytes, and further illustrated the biological significance of huoMTOC in physiology and pathology.

Spindle is a dynamic bipolar structure self-organized from microtubule and microtubule-associated proteins. This microtubule-based spindle mediates the chromosome transmission to daughter cells during cell division. Accurate spindle assembly is crucial for chromosome precise bi-orientation and segregation in both mitosis and meiosis. In somatic mitosis, duplicated centrosomes as the major MTOCs take responsibility for microtubule nucleation and spindle pole organization of the centrosomal spindle. And each centrosome contains a pair of centrioles surrounded by the pericentriolar material.

Conversely, canonical centrosomes are degraded in female meiosis of many species, such as *Caenorhabditis elegans*, *Drosophila*, mouse, human, and some other mammals. And the spindle microtubule should be organized in absence of centrosome. But so far investigations show that the mechanisms of meiotic spindle assembly between these species are not conserved.

## Different mechanisms of spindle assembly without MTOC structures in *C. elegans* and *Drosophila* oocytes

**Figure 1.**
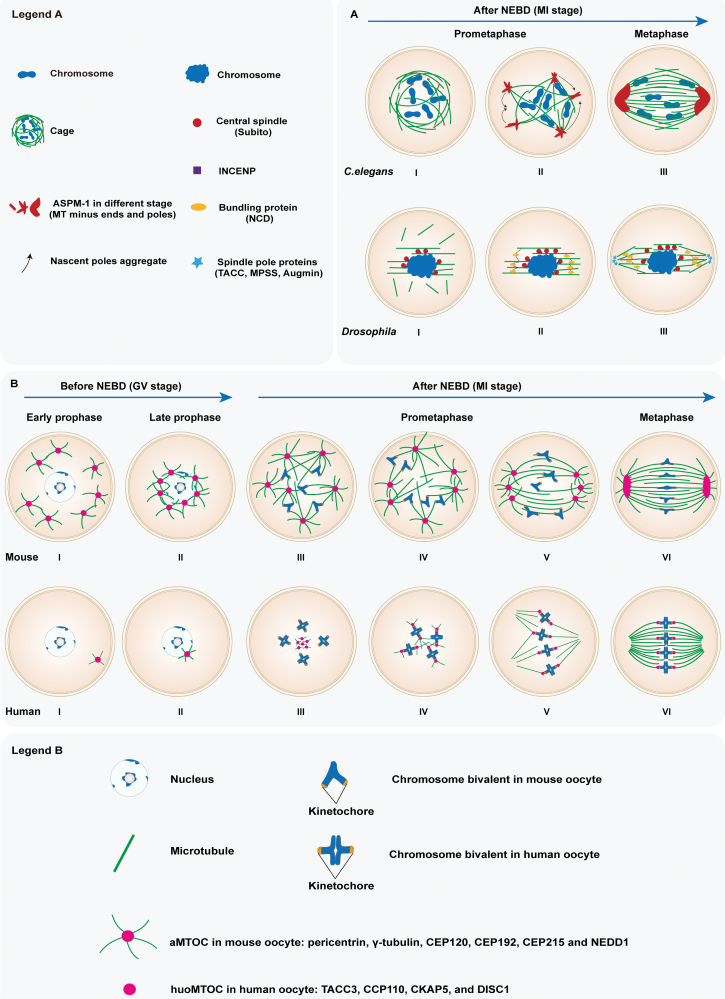
**Mechanistic models for acentrosomal spindle assembly in oocytes between different species.** (A) The dynamic processes of spindle assembly without MTOC structures in *C. elegans* and *Drosophila* oocytes. The different objects are indicated in legend A. (B) The dynamic processes of spindle assembly mediated by aMTOCs in mouse oocytes and huoMTOC in human oocytes. The aMTOCs and huoMTOC show multifaceted differences such as number, location, and constituent protein. The different objects are indicated in legend B. The detailed processes of spindle assembly are seen in the text.

Assembly of acentrosomal spindles in *C. elegans* oocytes occurs with no observed MTOC structures [1, 2]. In *C. elegans* oocytes, the meiotic spindle assembly is not driven via coalescence of MTOCs asters, but mainly proceeds through microtubule nucleation, sorting by motors and organization into poles [1]. After beginning with nuclear envelope breakdown (NEBD), microtubules initially form a “cage-like” structure adjacent to the nuclear envelope. While, ASPM-1 forms puncta at the ends of microtubules at the cage [1] (Fig. 1A, panel I, top). Then the microtubule minus ends of this cage structure is sorted to the periphery of the microtubule bundles by KLP-18 (kinesin-12)/MESP-1 (MESP-1) complex. Next, ASPM-1 foci are enriched on the ends of microtubules array and begin to form larger stretches connecting multiple microtubule bundles and forming the multiple nascent poles [1] (Fig. 1A, panel II, top). Finally, the multipolar spindle continuously coalesces until the steady bipolar spindle is established [1] (Fig. 1A, panel III, top). Besides, the KLP-7, a single mitotic centromere-associated kinesin/kinesin-13 member in *C. elegans*, maintains the spindle to a bipolar state by limiting both microtubule accumulation and pole number during oocyte meiotic spindle assembly [2].

Similarly, no formation of MTOCs has been reported in the *Drosophila* oocytes, though numerous studies have focused on how the bipolar spindles organize. Instead, these studies suggest a mechanism model that spindle assembly is guided by chromosomes, which recruit and/or nucleate the microtubules [3, 4]. As NEBD advances, the microtubules are assembled around the chromosomes into two kinds of fibers, the interpolar microtubules and kinetochore fibers [3]. The interpolar microtubules organize along the tangent of the chromosomes and then establish axis, which is called the central spindle. The initial assembly of microtubules around chromosome requires kinesin-6 protein Subito (SUB) [3], which can also recruit INCENP, a key subunit of chromosome passenger complex (CPC), to encourage the central spindle formation and stability by stimulating chromosome-microtubule interactions [4] (Fig. 1A, panel I, bottom). Subsequently, the interpolar microtubules begin tapering towards two poles, preparing for the establishment of bipolarity. Simultaneously, the kinetochore fibers are organized, bundled, and then attached to the kinetochores of the chromosomes by NCD protein, a microtubule motor (Fig. 1A, panel II, bottom). The interpolar and kinetochore microtubules crosslink, elongate, and taper to poles [3] (Fig. 1A, panels II–III, bottom). Moreover, Augmin, an 8-subunit complex, recruits the γ-tubulin complex for new microtubule nucleation and assembly on existing microtubules near the acentrosomal poles, which eventually assemble stable and bipolar meiotic spindle [3, 5]. And RanGTP also has a role in organizing the spindle poles through regulating proteins such as transforming acidic coiled-coil protein (TACC), minispindles, and the HURP homolog, Mars [3, 6] (Fig. 1A, panel III, bottom).

## The aMTOC-directed spindle assembly in mouse oocytes

In contrast, some vertebrate oocytes contain acentriolar microtubule organizing centers (aMTOCs) that functionally substitute centrosomes during acentrosomal spindle assembly.

The aMTOCs in mouse oocytes involve partial pericentriolar material components such as pericentrin, γ-tubulin, CEP192, CEP120, CEP215, and NEDD1 [7]. And the aMTOC-directed spindle assembly has been elucidated in mouse oocytes [8]. In the beginning, more than 80 aMTOCs self-organize from a cytoplasmic interphase-like microtubule network during early prophase arrest (Fig. 1B, panel I, top). Then aMTOCs are gradually recruited and move centripetally through direct interaction with the nuclear membrane until NEBD (Fig. 1B, panel II, top). Meanwhile, the chromosome clusters at nuclear envelope (Fig. 1B, panels I–II, top). Immediately after NEBD, aMTOCs are clustered at the center and mediate the enormous increase of surrounding microtubules mass in RanGTP-dependent manner, which mediate the individualization of clustered chromosomes at the same time (Fig. 1B, panel III, top). Next, the microtubule ball is formed and then transformed into multiple spindle poles by a series of aMTOC clustering events. Chromosomes are separated on the surface of the microtubule ball (Fig. 1B, panel IV, top). Through progressive clustering of multiple poles and activation of kinesin-5, multipolar aMTOCs assemble into a bipolar intermediate and which then elongates along the dominant axis. The chromosomes constantly stretch at the same time (Fig. 1B, panel V, top). Finally, stable barrel-shaped spindles with astral-like microtubules and aligned chromosomes are built on the equatorial plate in metaphase I (MI) oocytes (Fig. 1B, panel VI, top). In addition, a prominent “liquid-like spindle domain” which contains multiple centrosomal and microtubule-associated proteins can facilitate the spindle assembly in mouse oocytes [7]. This dynamic process of acentrosomal spindle assembly in mouse oocytes takes over 4 h.

Nevertheless, since the 1980s, prominent aMTOCs are not detected at the meiotic spindle poles in human oocytes through continuously numerous studies have shed light on human oocyte meiosis. Previously functional studies have supposed that chromosome and RanGTP mediate the spindle microtubule nucleation and subsequent organization [9], but it remains unclear how the spindle assembles exactly in female meiosis.

## The huoMTOC-directed spindle assembly in human oocytes

Recently, an aMTOC-like structure responsible for microtubule nucleation and meiotic spindle assembly was firstly identified in human oocytes by Wu et al [10]. Among numerous centrosomal/microtubule proteins localize to the spindle microtubules, four proteins—TACC3, CCP110, CKAP5, and DISC1 are found to be localized to both spindle microtubules and kinetochores in MI oocytes. Notably, each of the four proteins shows an unusual structure surrounded by microtubules in human germinal vesicle (GV) oocytes just before NEBD. This previously unknown and specific protein structure is named human oocyte microtubule organizing center (huoMTOC). Compared to aMTOC in mouse oocytes, the huoMTOC shows distinct characteristics in terms of number, localization, and constituent elements. The detailed dynamic process of huoMTOC regulating female spindle assembly is described below. Initially, one huoMTOC assembles near the cortex in the early prophase of GV oocytes (Fig. 1B, panel I, bottom). And then the huoMTOC gradually migrates to the nuclear envelope and further expands before NEBD (Fig. 1B, panel II, bottom). In the meanwhile, the nucleated microtubes surrounding the huoMTOC keep growing until the resumption of meiosis. At the early stage of NEBD, the huoMTOC localizes near the chromosomes and then becomes fragmented (Fig. 1B, panel III, bottom). The microtubules around huoMTOC are also disassembled and hardly observed. As the elapse of time, the fragmented huoMTOC is recruited to kinetochores and initiates microtubule nucleation for meiotic spindle assembly (Fig. 1B, panel IV, bottom). Through a transient multipolar spindle stage (Fig. 1B, panel V, bottom), eventually, the spindle converts into a bipolar structure. And the huoMTOC aggregates on kinetochores and the spindle in metaphase oocytes (Fig. 1B, panel VI, bottom). Furthermore, disruption of huoMTOC impairs spindle microtubule nucleation and spindle assembly. In particular, TACC3 depletion cause severe spindle defects and clinical oocyte maturation arrest.

Centrosomes elimination from oocytes during development is thought to ensure correct centriole number and proper mitosis upon fertilization and to prevent parthenogenesis. Although meiotic spindles lacking centrosomes in female oocytes is common, oocytes in various species have formed precise and specific mechanisms of spindle assembly, just reflecting the conservation and rich diversity of evolution. For example, RanGTP is essential for microtubule polymerization and spindle assembly in human oocytes [10], but spindles still assemble if RanGTP is inhibited in mouse or *Drosophila* oocytes [6], which suggesting RanGTP is not necessary for these two species. Furthermore, the spindle assembly is directed by chromosomes in both human and *Drosophila* oocytes, but the meiotic spindle assembly in human oocytes requires the huoMTOC [10], and that in *Drosophila* oocytes needs SUB and CPC [3]. The reasons for similarity and distinction about spindle assembly in different species need to be further investigated in the future.

As only for homo sapiens, oocyte spindle assembly takes a longer time and shows multipolar and instability, compared to the mouse and other species. These unique features may contribute to a high frequency of aneuploidy in human eggs. But for the scarcity of human oocytes, the detailed mechanisms of spindle assembly remain largely unknown. The study by Wu et al. suggests that a unique physiological mechanism for initiating microtubule nucleation and spindle assembly has evolved in human oocytes. This finding provides several compelling evidence to demonstrate that huoMTOC is responsible for the meiotic spindle assembly in human oocytes, and lays foundations for further study in mechanisms of spindle assembly.
